# AttendAffectNet–Emotion Prediction of Movie Viewers Using Multimodal Fusion with Self-Attention

**DOI:** 10.3390/s21248356

**Published:** 2021-12-14

**Authors:** Ha Thi Phuong Thao, B T Balamurali, Gemma Roig, Dorien Herremans

**Affiliations:** 1 Information Systems Technology and Design, Singapore University of Technology and Design, 8 Somapah Rd, Singapore 48737, Singapore; thiphuongthao_ha@mymail.sutd.edu.sg (H.T.P.T.); dorien_herremans@sutd.edu.sg (D.H.); 2 Science, Mathematics and Technology, Singapore University of Technology and Design, 8 Somapah Rd, Singapore 48737, Singapore; balamurali_bt@sutd.edu.sg; 3 Computer Science Department, Goethe University Frankfurt, 60323 Frankfurt, Germany

**Keywords:** neural networks, self-attention, emotion prediction, MediaEval 2016, COGNIMUSE, affective computing, multimodal fusion, computer vision

## Abstract

In this paper, we tackle the problem of predicting the affective responses of movie viewers, based on the content of the movies. Current studies on this topic focus on video representation learning and fusion techniques to combine the extracted features for predicting affect. Yet, these typically, while ignoring the correlation between multiple modality inputs, ignore the correlation between temporal inputs (i.e., sequential features). To explore these correlations, a neural network architecture—namely AttendAffectNet (AAN)—uses the self-attention mechanism for predicting the emotions of movie viewers from different input modalities. Particularly, visual, audio, and text features are considered for predicting emotions (and expressed in terms of valence and arousal). We analyze three variants of our proposed AAN: Feature AAN, Temporal AAN, and Mixed AAN. The Feature AAN applies the self-attention mechanism in an innovative way on the features extracted from the different modalities (including video, audio, and movie subtitles) of a whole movie to, thereby, capture the relationships between them. The Temporal AAN takes the time domain of the movies and the sequential dependency of affective responses into account. In the Temporal AAN, self-attention is applied on the concatenated (multimodal) feature vectors representing different subsequent movie segments. In the Mixed AAN, we combine the strong points of the Feature AAN and the Temporal AAN, by applying self-attention first on vectors of features obtained from different modalities in each movie segment and then on the feature representations of all subsequent (temporal) movie segments. We extensively trained and validated our proposed AAN on both the MediaEval 2016 dataset for the Emotional Impact of Movies Task and the extended COGNIMUSE dataset. Our experiments demonstrate that audio features play a more influential role than those extracted from video and movie subtitles when predicting the emotions of movie viewers on these datasets. The models that use all visual, audio, and text features simultaneously as their inputs performed better than those using features extracted from each modality separately. In addition, the Feature AAN outperformed other AAN variants on the above-mentioned datasets, highlighting the importance of taking different features as context to one another when fusing them. The Feature AAN also performed better than the baseline models when predicting the valence dimension.

## 1. Introduction

Emotions influence our well-being, how we interact with each other, our actions and our judgments [1]. Much of the media we encounter in our daily life, such as music and movies, is specifically designed to evoke an emotional reaction. The emotional influence of movies on viewers has been investigated in many psychology studies [1,2,3,4,5]. According to [6], scenes, music, as well as sounds in movies can elicit different emotions in viewers.

Developing an automatic system for predicting the emotions evoked in viewers is not only useful for psychologists, but also for producers in the film and advertising industries.

Many existing methods have been built to recognize the emotions of humans *in* videos [7,8,9,10,11,12,13,14]. These methods, however, do not predict the emotions that a person experiences when *watching* movies. Fewer studies [15,16,17,18] have focused on the latter, i.e., predicting the emotions of viewers based on movies themselves. This is the main goal of this work. More specifically, we propose a model for movie viewer emotion prediction using features extracted from video, audio, and movie subtitles.

Many studies on predicting the affective responses of movie viewers typically use extracted features from video and audio streams [15,18,19,20,21]. Those features are then fused by applying either early fusion or late fusion techniques without explicitly taking the relationship among these modalities into account. To tackle this problem, we previously proposed a preliminary model based on a deep-learning architecture with self-attention, called the AttendAffectNet model [22]. This network predicts the emotions of movie viewers by making use of the self-attention mechanism introduced in the Transformer model [23].

In natural language processing (NLP), we often see that, instead of using convolution or recurrence, the self-attention mechanism in the Transformer architecture is used to compute representations of the input and output of the model [23]. Since then, this mechanism has been applied to other tasks, including speech emotion recognition [24], music generation [25], and human action recognition [26]. Interestingly, this mechanism can take both temporal and spatial dependencies [23,27] of a sequence of inputs into account.

In our preliminary work [22], this mechanism was used to predict the emotions of movie viewers, in terms of arousal and valence as defined by the Circumplex model of affect developed by Russell [28]. In [22], two versions of the AttendAffectNet, including Feature AttendAffectNet and Temporal AttendAffectNet, were proposed. The former applies the self-attention mechanism to visual and audio features representing a whole movie excerpt, while the latter applies self-attention on the time domain. More specifically, in the Temporal AttendAffectNet, each movie excerpt is cut into equal parts. Visual and audio features are extracted from each movie part and fused before they are used as the model input.

The current paper is an extended version of our previous model [22]. In addition to visual and audio features, we also consider the text features extracted from movie subtitles. A combination of the Feature and Temporal AttendAffectNet models is also explored by applying the self-attention mechanism on both the extracted features as well as the time domain.

To have a comparable baseline, we also train and validate models using the long short-term memory structure (LSTM) and fully connected layers only from [21] using exactly the same inputs as those used for the proposed AttendAffectNet. We then compare their performance on two standard datasets, namely the MediaEval 2016 dataset for the Emotional Impact of Movies Task (EIMT16) [29] and extended COGNIMUSE [30,31].

**Figure 1:**
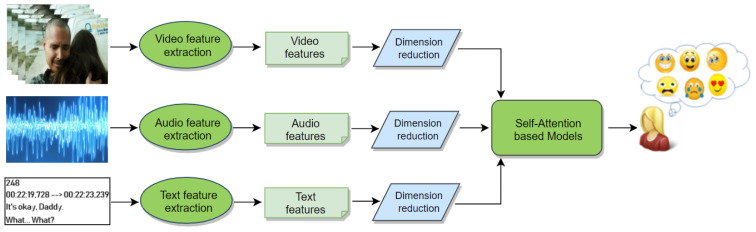
Overview of the proposed AttendAffectNet (ANN). Feature vectors are extracted from video, audio, and movie subtitles. We reduce their dimensionality before feeding them to the self-attention based models, which predict the affective responses of movie viewers.

Our self-attention-based approach is visualized in Figure 1. The input of our proposed AttendAffectNet includes visual, audio, and text features extracted by making use of pretrained deep convolutional neural network architectures (CNN) and other toolkits. More specifically, appearance features are extracted using the ResNet-50 network [32] as well as the RGB-stream I3D network [33].

The FlowNet Simple network (FlowNetS) [34] is used to extract motion features while VGGish [35] and the OpenSMILE toolkit [36] are used for audio feature extraction. In addition, the Bidirectional Encoder Representations from the Transformers model (BERT) [37] pretrained on Wikipedia & BookCorpus [38] is used to extract features from movie subtitles. All of these features are then passed to our AttendAffectNet model to predict the emotions of movie viewers.

Extensive experiments were conducted on the EIMT16 [29] and the extended COGNIMUSE dataset [30,31]. Note that, for the extended COGNIMUSE, the clips are cut into 5-s segments as in [19]. A careful analysis of the impact of video, audio, and movie subtitles on the accuracy of the emotion prediction task is performed.

We also investigate whether a combination of visual, audio, and text features could improve the performance of the proposed emotion prediction models. Our results show that audio features have a significant effect on driving the emotions of movie viewers, while movie subtitles are not as important as video and audio streams.

Additionally, we compare the performance of our AttendAffectNet model to the performance with only fully connected layers and LSTM as described in [21]. Overall, our proposed approach with self-attention performs better than the fully connected neural network and the LSTM when using the same feature vectors as the model inputs. The Feature AttendAffectNet model obtains higher accuracy compared with both the Temporal AttendAffectNet and Mixed AttendAffectNet on both datasets.

A possible explanation for this phenomenon could be that the duration of movie excerpts in these datasets is sufficient to deliver emotional content. Finally, the Feature AttendAffectNet also outperforms other baseline models for valence prediction. The source code of the AttendAffectNet together with pretrained models is available at https://github.com/ivyha010/AttendAffectNet (accessed on 7 December 2021).

An overview of the existing literature related to the self-attention mechanism and multimodal emotion prediction is given in the next section. In Section 3, we describe feature extraction techniques in detail. In Section 4, our proposed architecture is presented. Our experimental setup, together with the obtained results, is shown in Section 5 and Section 6, followed by our conclusions in Section 7.

## 2. Literature Review

Predicting emotional expressions of humans in videos has been studied by many researchers [39,40,41,42,43,44]. Some other research studies [15,21,30,45] have used videos to predict the affective responses of the viewers. Generally, however, this task has attracted little attention. Knowing which emotions are induced from videos has many applications, both in a commercial setting as well as in a research setting. Below, we offer an overview of the existing approaches for representing emotions, then we focus on multimodal representations, and finally we zoom in on multimodal emotion prediction models.

### 2.1. Emotion Representation Models

When it comes to representing emotions, many models have been proposed. In general, there are two major groups: dimensional (continuous) [46,47], and categorical (discrete) models [48,49]. The categorical approach [48] represents emotions as different discrete categories. For example, Ekman et al. [50] conducted research across multiple cultures and finally proposed a list of six basic emotions (including sadness, surprise, fear, disgust, anger, and happiness). More categories (such as amusement, contempt, and embarrassment.) [48] were later added to this list. In [51], 27 categories of emotion were identified based on 2185 short videos, which were used to induce specific emotions.

In [52], the Geneva Emotional Music Scales (GEMS) model was proposed to measure emotions driven by music. There are many versions of GEMS, such as the GEMS-9 and the GEMS-25. Notably, the GEMS-45 consists of 45 emotion terms, which can be divided into nine different categories. According to the dimensional approach, emotions are represented in a continuous way. Famous models that take this approach are the Vector model [53], the Positive Activation–Negative Activation model [54,55], and the Circumplex model [28,56].

In many affective computing studies [30,57,58,59,60,61,62], Russell’s Circumplex model of affect [28] is applied to represent human affective responses. According to this model, we can map human emotions into a space, which includes valence and arousal dimensions. The x-axis (valence) specifies how negative or positive the emotion is, while the y-axis measures the arousal of emotions from passive to active.

In addition to arousal and valence, there is also a third dimension called dominance in the circumplex model of affect [28]. This dimension indicates the degree of “attention” or “control” [63,64]. According to [31], however, dominance is often omitted due to being difficult to annotate. In this work, we use Russell’s Circumplex model with valence and arousal dimensions.

### 2.2. Multimodal Representations for Emotion Prediction

In movie analysis, it is common to consider different modalities including video, audio, and text [65]. In what follows, we discuss the existing research on models that incorporate one or more of these modalities into their affective response prediction.

#### 2.2.1. Video Modality

Many research papers [13,66] leverage the representational power of deep CNNs to extract visual features from the video stream to recognize the emotions of humans appearing in videos. To extract features from human faces appearing in videos, the VGG16-Face model, which is the VGG16 network [67] fine-tuned on the FER2013 face emotion dataset [68], is utilized in [13]. In addition to the VGG16-Face network, the 3-dimensional convolutional network (C3D) has also been applied to simultaneously model the appearance and motion of objects in videos [13].

Typically, CNNs are used to extract features from video frames to predict the emotions of movie viewers [16,17,21,45,69,70]. Apart from the appearance features obtained from still frames, motion features extracted from optical flow [71] are also important in many tasks including action recognition [72,73,74], facial expression recognition [75,76,77], as well as emotion prediction of movie viewers [21,45,69].

In many studies on action recognition, the appearance and action information can either be extracted simultaneously using C3D [78,79,80], or it can be processed separately, for instance as two-stream CNNs [72,81], or as the two-stream inflated 3D CNN (I3D) [33]. These approaches are employed in models for emotion prediction of video viewers (C3D in [17,18], I3D in [45]) and emotion recognition (C3D in [13]).

In our previous work [22] on predicting the emotions of movie viewers, we applied the pretrained ResNet-50 [32] and RGB-stream I3D [33] networks on still RGB frames to extract appearance features of objects. Although combining optical flow with RGB increases the accuracy of models for action recognition [33,72] and emotion prediction [21], the cost of estimating the optical flow is high.

To balance the cost and efficiency, we therefore use the pretrained I3D model with only its RGB input stream (i.e., RGB-stream I3D) to extract appearance features from successive frames. The simultaneous use of both ResNet-50 and RGB-stream I3D networks was also explored in [82] for action detection. According to [21], motion features are also important for emotion prediction. However, rather than extracting motion features from optical flow as done in [21,82], we make use of the FlowNetS network pretrained on the Flying Chairs dataset [34] to directly extract motion features.

#### 2.2.2. Audio Modality

According to many studies [52,83,84,85,86], emotions are highly influenced by music/speech; hence, it is important not to exclusively focus on video frames when doing emotion prediction. The emotional content of a dialogue is determined by both the tone and the content of spoken words as well as the semantic context of the movie scene and possible background music [87].

In addition to neural network architectures, such as SoundNet [88], VGGish, Inception, ResNet and AlexNet [35], we can use toolkits, such as OpenSMILE [36] and YAAFE [89] for audio feature extraction. Audio features obtained by using the OpenSMILE toolkit, are proven to be effective for the emotion prediction task [18,21,61]. In [90] models using VGGish-extracted features outperform those that use features extracted by applying SoundNet, and the OpenSMILE toolkit. A combination of audio features obtained by using both OpenSMILE and VGGish provided good prediction accuracy in our previous work [22]. Therefore, these features are also utilized in this work.

#### 2.2.3. Text Modality

In addition to audio and video, it has been shown that text data is useful when creating a good emotion and sentiment analysis model [91]. According to [92,93], the semantic analysis of movie subtitles can improve affect classification. Video subtitles have been used to develop an emotion recognition model for soap opera episodes [94], for the emotional analysis of movies [95], as well as to detect emotional scenes from movies [87]. Many of the current studies on multimodal emotion prediction [15,19,30,45], however, have focused on using visual and audio features without including the subtitles. In this study, in addition to video and audio streams, we explore the potential of using movie subtitles to build an affective representation of movies, which is then utilized to predict the emotions of movie viewers.

Word embeddings were first introduced in [96]. They are also referred to as distributed word representations [97], in which words are represented as low-dimensional vectors that contain semantic and syntactic information [98]. Syntactic information refers to the structural role of words, while semantic information refers to the meaning of words [98]. Word embeddings are generally grouped into two categories: frequency-based embeddings and pretrained embeddings [99,100]. Some popular frequency-based embedding techniques include Bag-of-Words, and Term Frequency–Inverse Document Frequency (TF-IDF) [101].

Common pretrained word embedding techniques include Word2Vec [102,103], GloVE [104], and fastText [105]. Word embedding representations are often used as input to models that tackle NLP tasks, such as sentiment analysis (e.g., a CNN with Word2vec and GloVE in [106], a CNN with Word2Vec in [107]), text classification (e.g., Support Vector Machines (SVM) [108] accompanied with Word2Vec, TF-IDF in [109]), question answering (e.g., a dependency-tree recurrent neural network with Word2Vec in [110]), text generation (e.g., Generative Adversarial Networks with Word2Vec in [111]).

Instead of using pretrained word embedding techniques to represent individual words, context-based representations can be used to create vectors that represent whole *sentences*. Context-based representations take the order of words as well as the set of co-occurring words into account and compute a vector for the whole sentence.

There are many papers offering pretrained contextual representations, such as Semi-supervised Sequence Learning [112], Generative Pretrained Transformer (OpenAI GPT) [113], ELMo (Embeddings for Language models) [114], Universal Language Model Fine-tuning (ULMFiT) [115], and BERT [37]. In these models, word and sentence representations are learned such that they best capture underlying meanings and relationships.

The Semi-Supervised Sequence Learning model in [112] and OpenAI GPT [113], however, are unidirectional (i.e., only words to the left or right are used to contextualize each word). In both ELMo and BERT, the text representations are learned by using a bidirectional language model. A concatenation of both left-to-right, as well as right-to-left language models trained independently is utilized in ELMo.

The BERT model, on the other hand, uses a deep neural network. Inspired by the idea of the Transformer [23], the BERT model is considered as one of the notable achievements in NLP. In the BERT model, masked language models are applied to obtain pretrained deep bidirectional representations from unlabelled text, in which the left context is joined with the right one in all layers.

According to [37], the pretrained BERT model can be fine-tuned to tackle many other tasks, simply by adding the appropriate output layer. We can also utilize the BERT model for feature extraction, and thus, the pretrained BERT model can be applied to obtain contextual embeddings, which are then passed to models performing other tasks. The BERT model is applied and performs well in sentiment analysis [116,117,118]. In this study, we use BERT [37] pretrained on Wikipedia & BookCorpus [38] to extract contextualized word embeddings from movie subtitles and use them as the input to our proposed AttendAffectNet.

### 2.3. Multimodal Emotion Prediction Models

When implementing a multimodal fusion approach, features are extracted from various modalities (such as audio, video, text) and fused to perform a task. This approach is used in many research problems including semantic video analysis [65], emotion recognition [119], visual question answering [120], cross-modal retrieval task [121], etc. It is also a common approach in recent studies on emotion recognition of humans in videos [66,122,123] and on evoked emotion prediction from movies [15,19,21], in which features extracted from video and audio are used.

Often, features extracted from different modalities are combined by applying multimodal fusion schemes, such as early or late fusion [65]. In particular, early fusion is used when modalities are fused in the feature space, i.e., unimodal features are combined before being used to learn concepts. In late fusion, modalities are combined in the semantic space, i.e., unimodal features are first transformed to separately learned concept scores, which are then combined to learn concepts [65]. Aside from these two fusion techniques, we can also consider collaborative fusion [124,125,126], which is used to encourage collaboration among multiple modalities.

Many unsupervised and supervised approaches have been developed to build multimodal models for emotion prediction. Support vector regression models (SVR) have been applied to the task of emotion prediction of movie viewers on the Discrete LIRIS-ACCEDE dataset [60]. In the SVR models, various visual and audio features are used to separately predict valence and arousal values. Hidden Markov models (HMMs) are trained using low-level features obtained from audio and video to predict valence and arousal values in frames on the extended COGNIMUSE in [30,31].

A follow-up study in [21] further improved the emotion prediction accuracy, in which a neural network consisting of fully connected layers only was used instead of HMMs. In [21], features carrying the information on the appearance and motion of objects appearing in the video, as well as audio features are obtained by using the pretrained ResNet-50, ResNet-101 networks, and the OpenSMILE toolkit, respectively.

The prediction accuracy of the fully connected network is compared to a two-layer LSTM model that uses the same feature sets. The former model outperforms the latter on the extended COGNIMUSE dataset. In other studies [15,19] on this dataset, the authors cut movie clips into 5-s excerpts. The values of valence and arousal are then averaged across all frames in each excerpt.

In [19], these average arousal and valence values are predicted for every 5-s excerpt by using a simple linear regression together with fusion schemes involving early fusion, late fusion, and the mixture of experts (MoE) fusion. The MoE fusion performs better than the two previous ones. A follow-up study in [15] uses an LSTM network and correlation-based feature selection [127].

This model obtains a higher prediction accuracy compared to the previous model proposed in [19]. In [45], an adaptive fusion recurrent network is built based on an LSTM. This approach obtains higher performance than the Random Forest model [17], SVR [17,18] as well as the LSTM and Bidirectional-LSTM [20], and the arousal-valence discriminant preserving embedding algorithm [16] on the EIMT16 dataset [29].

While LSTM models take the long-term dependencies into account when modeling sequential data, they do not capture the spatial dependencies among inputs. The recently developed Transformer model [23], on the other hand, could overcome this disadvantage by using the self-attention mechanism. The Transformer model outperforms the LSTM in various tasks involving speech emotion recognition [24] and text translation [128]. It also achieves good performance in the human action recognition task [26].

Inspired by these findings, we use the Transformer structure in this study for the task of evoked emotion prediction from movies. This mechanism may capture the correlation among multiple modalities as well as between temporal inputs. In addition, the performance of our proposed models is compared to that of the LSTM model and the fully connected network proposed in [21]. We use the EIMT16 as well as the extended COGNIMUSE dataset to train and validate our proposed models.

Our multimodal approach for emotion prediction of movie viewers will be discussed in detail in the next section. The techniques that are applied to obtain the representation of movies will be described first. Then we move on to a description of our proposed self-attention based architecture.

## 3. Multimodal Feature Extraction

Our proposed emotion prediction models include three modalities: video, audio, and text (movie subtitles). In the following subsections, we provide details on how the features from each of these modalities are extracted.

### 3.1. Visual Features

The appearance of objects and their motion in each video scene can induce different kinds and degrees of emotion in viewers [6]. We obtain the information on the appearance of objects by applying pretrained networks (as discussed below) on RGB frames. In addition, we also extract motion features from successive frames. Since videos might have different length and frame rates, prior to extracting visual features, a fixed number of frames (denoted as *T*) are extracted from each video. Particularly, for every $\frac{t_{i}}{T}$ seconds, only one frame is extracted, whereby $t_{i}$ is the length (in seconds) of the *i*-*th* clip. Extracting a predetermined number of frames from clips is also performed in [26,82].

**Appearance features** The ResNet-50 network [32] pretrained on the ImageNet dataset [129] is first used to obtain the static appearance features of objects. In particular, we pass every RGB frame through the pretrained ResNet-50 network (with the exception of its final fully connected layer) to obtain a 2048-feature vector from each frame. Element-wise averaging is then performed on the extracted features across all *T* frames (from each movie excerpt) to finally obtain a vector of 2048 features.

In the RGB-stream I3D network [33], the spatio-temporal features are learned directly from a stack of successive RGB frames by employing 3D convolutions and 3D pooling operations. This also forms a solid baseline for the human action recognition task. Therefore, we use the Inception-v1-based I3D model [130], which is pretrained on the Kinetics dataset [131], to obtain spatio-temporal features. Particularly, we pass *T* frames from each clip to the RGB input stream of the pretrained Inception-v1-based I3D network, except for the layers after its “mixed-5c” one. This provides a feature map of size $1024\times \frac{T}{8}\times \frac{H}{32}\times \frac{W}{32}$, where *H*, *W* are the frame height and width, respectively. Average pooling with a kernel size of $\frac{T}{8}\times \frac{H}{32}\times \frac{W}{32}$ is then applied on this feature map to finally obtain a 1024- dimensional feature vector.

**Motion features** Due to the high computational cost in optical flow estimation, instead of estimating optical flows and extracting motion features from them, we make use of the FlowNetS network [34] to obtain low-level motion features. This network is pretrained on the Flying Chairs dataset [34], and its structure includes two parts: contracting and expanding. The contracting part is applied as the motion feature extractor in this study. In particular, each pair of successive frames is fed to the contracting part to obtain a 1024-feature vector. The motion features extracted from all (T−1) pairs of frames (from each movie excerpt) are element-wise averaged to finally obtain a vector of 1024 features.

### 3.2. Audio Features

The OpenSMILE-extracted features as well as those obtained by using the pretrained VGGish neural network [35] have shown to provide meaningful input for the emotion prediction task [21,90]. We therefore chose to include those audio features as part of our proposed model inputs.

**VGGish model** The VGGish neural network with parameters pretrained on the AudioSet dataset [132] for sound classification is used to extract audio features. In the preprocessing step, the audio from each movie clip is first split into non-overlapping 0.96-s frames. Then, a spectrogram is computed using the short-time Fourier transform for each 0.96-s frame, whereby the window size and the hop size are 25 and 10 ms, respectively. After that, each spectrogram is mapped to 64 Mel bins to compute a Mel spectrogram before a logarithmic operation is applied to obtain the log Mel spectrogram of size 96 × 64 for each segment. The log Mel spectrogram is then passed to the pretrained VGGish model, which includes six convolutional layers followed by two fully connected layers. This results in a 128-dimensional audio feature vector for each 0.96-s audio segment. The 128-feature vectors extracted from all audio segments (from each movie excerpt) are element-wise averaged to obtain a 128-dimensional vector of features.

**OpenSMILE-extracted features** A 1582 feature vector (including low-level descriptors, such as intensity, pitch, loudness, MFCCs, as mentioned in [133]) is extracted by using the “emobase2010” configuration file proposed in the INTERSPEECH 2010 paralinguistics challenge [134]. Note that the used audio frame and the hop size are 320 and 40 ms, respectively. We then compute an element-wise averaging of those feature vectors across all 320 audio frames (from each movie excerpt) to obtain a 1582-dimensional feature vector.

### 3.3. Text Features

The BERT-Base network (including 12 encoder layers, 12 attention heads with a feed-forward network of 768 hidden units each) [37] pretrained on Wikipedia & BookCorpus [38] is used to extract contextual embeddings. During the pretraining process, the last encoder layer of the model is customized for the masked language model and the next sentence prediction task in [37], we therefore remove it from the pretrained BERT model when applying it to our task. As a result, we obtain a vector of 768 features from each token. Average pooling is then applied across all contextual embeddings (from each excerpt) to obtain a 768-feature vector.

## 4. Proposed AttendAffectNet Model

Inspired by the Transformer model [23], we propose the AttendAffectNet (AAN) model–a multimodal neural network that integrates the self-attention mechanism [23] to predict the emotions of movie viewers represented in valence and arousal dimensions using features extracted from video, audio, and subtitles. We implement and evaluate three variants of our proposed model. In the first variant, called Feature AAN, the self-attention mechanism is applied to the features obtained from different modalities. The second one is the Temporal AAN, in which the self-attention mechanism is applied to the movie time domain. The third one is the Mixed AAN, which is a combination of the previously proposed two variants. Before describing our proposed models in detail, we first take a quick look at the Transformer architecture.

**Transformer architecture** In the Transformer architecture, the positions of a sequence are related using the self-attention mechanism [23]. This is efficiently performed, not by using the original inputs directly, but by first projecting the inputs to queries, keys, and values, which are denoted as Q, K, and V, respectively. Their dimensions are $d_{q}$, $d_{k}$, and $d_{v}$, respectively, whereby $d_{k}$ is equal to $d_{q}$. This is done by applying fully connected layers many times (*h* times, which is also known as *h* heads).

The scaled dot-product attention (i.e., the dot-product attention scaled at $\frac{1}{\sqrt{d_{k}}}$) [23] is then conducted to obtain $d_{v}$-dimensional output vectors. Note that in the Transformer, the order of elements in a sequence is taken into consideration, whereby, the positional encodings are added to the embedding inputs as well as the previous outputs.

Similar to sequence translation models [135,136,137], the Transformer has an encoder–decoder structure. The input sequence is encoded into a sequence of continuous representations by a stack of encoders. The sequence of outputs is then generated by a stack of decoders, whereby, at each time step, only one output is created. The outputs generated at the previous steps are also used as part of the model input to create the subsequent one. For more information, we refer readers to the original paper [23].

Motivated by the self-attention mechanism in the Transformer architecture, we propose three variants of the self-attention-based model for emotion prediction of movie viewers.

### 4.1. Feature AttendAffectNet Model

**Figure 2:**
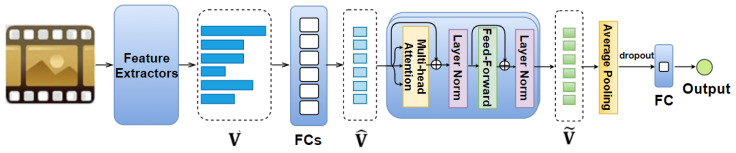
Our proposed Feature AttendAffectNet. For dimension reduction, the set of feature vectors V is fed to fully connected layers with eight neurons each (so as to obtain a set of dimension-reduced feature vectors $\hat{V}$) before being passed through *N* identical layers (each layer includes a multi-head self-attention accompanied with a feed-forward layer). The output of such stack is a set of encoded feature vectors Ṽ, which are then fed to an average pooling layer, dropout, and a fully connected layer (consisting of one neuron) to obtain the predicted arousal/valence values.

Previous studies [21,30,66,92,93] have shown that the use of different features is critical for achieving high accuracy in emotion prediction and emotion classification tasks; and that each feature plays a distinctive and important role in emotion prediction. Motivated by these findings, we propose the Feature AAN model (illustrated in Figure 2), in which the self-attention mechanism is applied to the visual, audio, and text features of the entire movie.

In this model, each feature vector ${\vec{v}}^{f}$ extracted from the movie excerpt (where f∈F— a set of all feature types mentioned in Section 3) is fed to an eight-neuron fully connected layer so as to obtain a dimension-reduced feature vector ${\vec{\hat{v}}}^{f}$. The sets of extracted feature vectors and the corresponding dimension-reduced ones are denoted as V and $\hat{V}$, respectively. We then feed the set $\hat{V}$ to *N* identical layers, whereby each of them includes two sub-layers: a multi-head self-attention followed by a feed-forward layer. Each of these sub-layers is enclosed by a residual connection [32] accompanied with a layer normalization [138]. The number of layers and heads used in this work is discussed in Section 5.2. Note that the order of vectors ${\vec{\hat{v}}}^{f}$(f∈F) is not considered in this model. The output of these *N* identical layers is a set **Ṽ** of encoded feature vectors ${\vec{ṽ}}^{f}$(f∈F), each of them includes eight elements. We then obtain an eight-dimensional vector by applying an average pooling to these encoded feature vectors. We also perform dropout along with a fully connected layer on this vector to obtain the final output.

### 4.2. Temporal AttendAffectNet Model

In this model, the time domain of movie excerpts is considered. For doing so, we first cut each movie excerpt into *n* equal parts (i.e., segments), from which visual, audio, and text feature vectors are extracted. The feature vector of type *f* (f∈F) extracted from the *s*-th movie part (s∈S={1,...,n}) is denoted as ${\vec{v}}_{s}^{f}$.

We pass feature vectors ${\left\{{\vec{v}}_{s}^{f}\right\}}_{s\in S}^{f\in F}$ to fully connected layers consisting of eight neurons each. This allows us to obtain the corresponding feature vectors ${\left\{{\vec{\hat{v}}}_{s}^{f}\right\}}_{s\in S}^{f\in F}$. All of the feature vectors that are obtained from the same *s*-th movie part are then concatenated together to obtain a representation vector ${\vec{u}}_{s}=\mathrm{concat}\left({\left\{{\vec{\hat{v}}}_{s}^{f}\right\}}^{f\in F}\right)$. Note that vector ${\vec{u}}_{s}$ consists of 8×|F| elements (where the size of the set F is denoted as |F|).

We denote the position of the *s*-th part as ${\vec{\mathrm{PE}}}_{s}\in R^{d}$, which is encoded by using Equation (1).

(1) $${\mathrm{PE}}_{s}^{\left(i\right)}=\left\{\begin{array}{cc} \sin\left(\omega_{k}.s\right) & \mathrm{if}\;i=2k\\ \cos\left(\omega_{k}.s\right) & \mathrm{if}\;i=2k+1, \end{array}\right.$$

in which $\omega_{k}=\frac{1}{{10,000}^{2k/d}}$; *d* is the size of vector ${\vec{u}}_{s}$; i=0,⋯,d. To take the order of movie parts into account, we add vectors ${\vec{\mathrm{PE}}}_{s}$ to vectors ${\vec{u}}_{s}$ to obtain a sequence of vectors ${\vec{u}}_{s}+{\vec{\mathrm{PE}}}_{s}$ (s∈S). This sequence is then fed to *N* identical layers, in which each layer contains a masked multi-head attention accompanied with a multi-head attention, and a feed-forward layer.

Using the masked multi-head attention, the current positions are kept from interacting with the future ones. In this model, the multi-head attention plays the same role as the one described in the Feature AAN model, except for the fact that its queries *Q* comes from the previous sub-layer (We refer the readers to Section 3.2.3 in [23] for more details). In both the Feature AAN and Temporal AAN, each feed-forward layer includes a ReLU activation accompanied with a fully connected layer of eight neurons. Similar to the Feature AAN, this model also uses the residual connection as well as the layer normalization [138].

**Figure 3:**
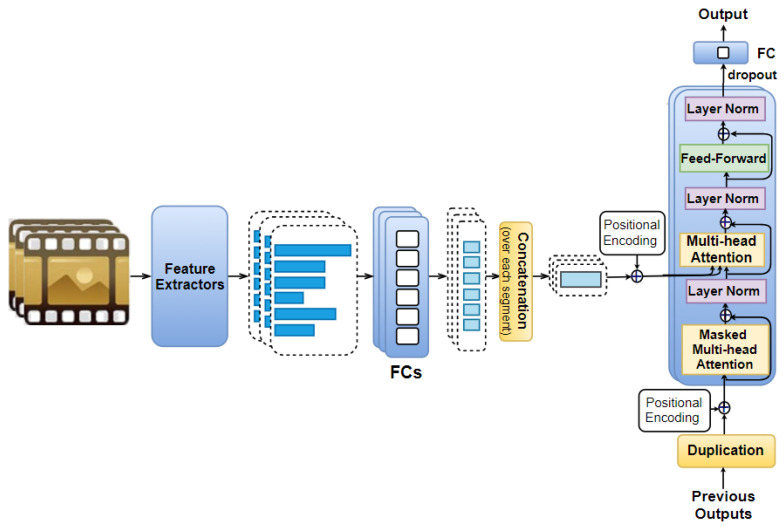
Our proposed Temporal AttendAffectNet: Feature vectors extracted from each movie part are passed to fully connected layers for dimension reduction before being combined together to create a representation vector (for each movie part). A positional encoding vector is added to this representation vector, which is then passed to *N* identical layers (each of them includes a mask multi-head attention, a multi-head attention accompanied with a feed-forward layer) followed by dropout and a fully connected layer consisting of only one neuron. We also add the positional encoding vectors to the previous outputs before using them as an additional input to the model to predict the subsequent output.

We also include dropout and a fully connected layer consisting of only one neuron like in the Feature AAN to obtain a scalar model output (i.e., predicted valence/arousal value) corresponding to each movie part. Note that $|{\vec{u}}_{s}|$ is 8×|F|, therefore, each previous scalar output is duplicated 8×|F| times to match the size of ${\vec{u}}_{s}$. We also tried a fully-connected layer of 8×|F| neurons instead of duplication, however, the model with duplication performs better. The positional encoding vectors are added to the previously duplicated outputs, which are then used as part of the model input to predict the next output as illustrated in Figure 3.

### 4.3. Mixed AttendAffectNet Model

**Figure 4:**
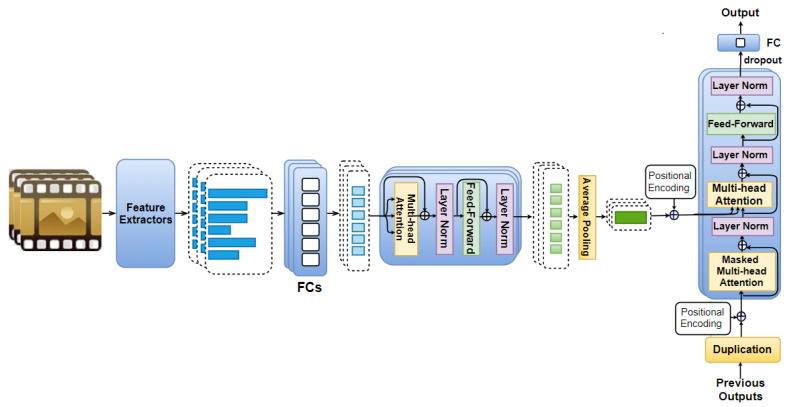
Our proposed Mixed AttendAffectNet: Feature vectors extracted from each movie part are first fed to fully connected layers for dimension reduction before being passed to *N* identical layers. Each of them includes a muli-head attention followed by a feed-forward layer. We apply average pooling to the outputs of those identical layers to obtain representation vectors corresponding to movie parts. We add positional encodings to these representation vectors, which are then fed to another set of *N* identical layers. These layers are similar to the previous ones, except that each of them includes one more layer called masked multi-head attention. This set of *N* identical layers is followed by dropout, and a fully connected layer. The previous outputs together with their corresponding positional encodings are used as the additional input to this model.

Combing the ideas from the two previous discussed model variants, a third variant, namely Mixed AAN (illustrated in Figure 4), is also explored in this work. In the Mixed ANN, self-attention is first used for feature vectors, which are obtained from the same movie part as described in the Feature AAN. Then, we also apply it on the time domain as described in the Temporal AAN.

Each movie clip is cut into *n* equal parts, from which visual, audio, and text features are extracted. We denote these extracted vectors as ${\left\{{\vec{v}}_{s}^{f}\right\}}_{s\in S}^{f\in F}$ as mentioned in Section 4.2, which describes the Temporal AAN. Feature vectors ${\left\{{\vec{v}}_{s}^{f}\right\}}^{f\in F}$ (extracted from the same *s*-th movie part) are fed to fully connected layers of eight neurons each (to obtain vectors ${\left\{{\vec{\hat{v}}}_{s}^{f}\right\}}^{f\in F}$), followed by *N* identical layers (as described in Section 4.1 for the Feature AAN) to obtain the encoded feature vectors ${\left\{{\vec{ṽ}}_{s}^{f}\right\}}^{f\in F}$ of eight elements each. This is followed by average pooling to obtain an eight-element output vector $\vec{{ṽ}_{s}}$. The positional encoding vector ${\vec{\mathrm{PE}}}_{s}$ is also added to every eight-element output vector $\vec{{ṽ}_{s}}$ before being fed to *N* identical layers as described in the Section 4.2 on the Temporal AAN.

Similar to the Temporal AAN, the previous outputs together with their corresponding positional encoding vectors are then used as an additional input to this model to predict the subsequent output. We also adopt the dropout and a one-neuron fully connected layer in this model to obtain the predicted arousal/valence.

Note that, in the Mixed AAN, the previous outputs are scalar (i.e., valence/arousal values); therefore, each of them is also duplicated eight times (instead of 8×|F| as proposed in the Temporal AAN) to force them to have the same size as the eight-element output vector resulting from the average pooling layer.

## 5. Experimental Set-Up

The performance of our proposed multimodal models on the task of predicting movie viewers’ emotions is assessed in a set of experiments. The experimental setup andh the datasets used are described below.

### 5.1. Datasets

We use two datasets, including the extended COGNIMUSE [30,31] and the EIMT16 dataset [29], to evaluate our proposed models.

#### 5.1.1. Extended COGNIMUSE Dataset

The extended version of the COGNIMUSE dataset [30] includes twelve movie clips with a duration of 30 min each and a frame rate of 25 frames per second. Seven of these movie clips belong to the COGNIMUSE dataset [31]. Emotion, represented in terms of arousal and valence, is annotated for each frame. The values of valence and arousal vary between −1 and 1.

This dataset provides both intended as well as experienced emotions, which are annotated by expert and non-expert movie viewers, respectively. The former represents the intention of the filmmakers, while the latter describes the emotion of viewers while watching movies. Since the inter-annotator agreement in the experienced emotion annotations is low [31], only the intended emotion annotations are used in studies [15,19,30].

Since arousal and valence values do not significantly change between successive frames in this dataset, in [15,19], the authors cut the movie clips into 5-s excerpts. The intended emotion corresponding to each excerpt consists of a pair of averaged valence and arousal values computed across all frames, i.e., only one pair of resulting valence and arousal values is obtained for each 5-s movie excerpt. Those intended emotion annotations are also used in this work.

#### 5.1.2. EIMT16 Dataset

The EIMT16 is part of the LIRIS-ACCEDE database (https://liris-accede.ec-lyon.fr/, accessed on 10 January 2019). It consists of two subsets: Global EIMT16 and Continuous EIMT16. The former was created for the task of predicting the emotion (valence/arousal) of movie viewers while watching short movie excerpts, whereby only one pair of arousal and valence values is annotated for each movie excerpt.

The latter is for predicting arousal/valence continuously on each second of the long movie clips. The aim of creating the extended COGNIMUSE dataset was similar to that of the Continuous EIMT16 dataset. In addition, compared to other datasets in the LIRIS-ACCEDE database, the number of movie excerpts in the Global EIMT16 is the highest. For those reasons, the Global EIMT16 is considered as a complement to the extended COGNIMUSE, and both of them are used in this study.

According to [29], there are 11,000 movie excerpts in the Global EIMT16. Each of them lasts between 8 to 12 s and is annotated with a pair of arousal/valence values, which vary between 0 and 5. The emotion in this dataset represents the expected values of the invoked emotion annotated by movie viewers.

### 5.2. Implementation Details

#### 5.2.1. Data Preprocessing

For the Global EIMT16 dataset, movie excerpts have various frame rates and durations; hence, a fixed number of frames (64 frames) are obtained from each excerpt by using the FFMPEG tool (https://www.ffmpeg.org/, accessed on 02 February 2020), wherein we set the frame rate to $\left\lceil \frac{64}{t_{i}}\right\rceil$ ($t_{i}$ is the *i*-th clip’s length). The extracted frames are then center-cropped to the size of 224×224 each.

We also apply this technique on the extended COGNIMUSE dataset, except for the fact that instead of extracting 64 frames, we use the entire set of frames obtained from each 5-s movie excerpt (i.e., 125 frames). Note that all 5-s movie excerpts in this dataset have the same frame rate (25 frames per second). The subtitles are only available for the extended COGNIMUSE dataset and they are not provided in the Global EIMT16 dataset, therefore, experiments on text features are conducted on the extended COGNIMUSE dataset only.

In the extended COGNIMUSE dataset, 5-s movie excerpts cut from each movie clip are provided in temporal order, therefore, each of them is considered as a component in the input sequence of the Temporal AAN as well as the Mixed AAN model. As the Global EIMT16 includes excerpts cut from various movies, and they are not consecutive. We therefore split each of them (with a length varying between 8 and 12 s) into 4 smaller parts, which are non-overlapping and annotated with the same arousal/valence values when conducting experiments for the Temporal AAN and Mixed AAN models.

Due to their relatively short length (2–3 s), we extract only 16 frames from each of them using the FFMPEG tool, instead of 64 frames like in the whole movie excerpt.

#### 5.2.2. Training Details

In this study, arousal and valence values are predicted using separate models. The Adam optimizer together with the following loss function *L* is used in the training phase:

(2) $$L=\mathrm{MSE}+\left(1-\mathrm{PCC}\right),$$

where MSE is the mean squared error between the predicted arousal/valence values and their ground truth, and the PCC is their Pearson correlation coefficient.

In this study, in addition to the OpenSMILE toolkit, we use pretrained deep neural networks (ResNet-50, RGB-stream I3D, FlowNetS, VGGish, and BERT) as feature extractors. Each extractor allows the input (i.e., image, audio, and movie subtitles) to propagate forward and to stop at a predetermined layer, whereby its output forms our extracted feature vector. One of the advantages of this approach is that we can obtain the robust and discriminative feature vectors learned by these deep neural networks without concerns about the memory usage and computational time, as is the case when fine-tuning or training these entire networks or some of their layers together with the self-attention layers.

Training the whole model, including the layers of these networks together with self-attention layers, is extremely computationally expensive due to the high number of trainable parameters, and might be prone to overfitting. For these reasons, we first apply the aforementioned deep neural networks as feature extractors, and then train the rest of our proposed AttendAffectNet model only. The computational complexity of the multi-head attention with linear transformations is $O(n^{2}\times d+n\times d^{2})$, where *n* is the length of the input sequence, and $d_{q}=d_{k}=d_{v}=d/h$ for each of the *h* heads.

In our experiments, to extract features, the average running time on each 5-s movie excerpt from the extended COGNIMUSE dataset is 0.01183 s for the pretrained ResNet-50 used to extract appearance features, 0.05449 s for extracting features from the RGB stream of the I3D network, 0.89369 s for FlowNetS, 0.12213 s for VGGish, and 9.20296 × 10^−7^ s for BERT, when performed on an NVIDIA GTX 1070 card with a 48 GB RAM. Note that the feature extraction can be done in parallel.

Using the extracted feature vectors as the input to our proposed AttendAffectNet, we only train the AttendAffectNet part of the model. To optimize the model, different experiments were performed to set the hyperparameters. From these experiments, we noticed that the computational cost rises and overfitting occurs when a larger number of heads are used. The proposed models obtain the highest performance when we use two heads.

The maximum number of epochs is 500, and the learning rate and the dropout rate are 0.0005 and 0.1, respectively, when training the Feature AAN. These hyperparameters are set to 1000, 0.001, and 0.5, respectively, for the Temporal AAN model. For the Mixed AAN, the maximum number of epochs is 500, the dropout rate is set to 0.5, and the learning rate is 0.01. For both the Temporal AAN and Mixed AAN models, the sequence length is fixed to 5 for the extended COGNIMUSE, and 4 for the Global EIMT16. For each of the three models, the batch size is set to 30, and the “patience” argument for the early stopping is set to 30 epochs.

The training time of our proposed AttendAffectNet is arguably fast. The average running time for each epoch is 6.64224 s, 7.81317 s, and 9.57832 s for the Feature AAN, Temporal AAN, and Mixed AAN, respectively. This is for a model that uses all visual, audio, and text features are used as input, a batch size of 30 and when the early stopping procedure is applied. All experiments are carried out using Python 3.6 on an NVIDIA GTX 1070 card with 48 GB RAM.

For the Temporal AAN and Mixed AAN models, the training is parallelized. During the training process, the previous outputs are available and they are used as an additional part of the model input to predict the subsequent ones. However, at the beginning of the evaluation phase, the whole model outputs are not provided, hence we must predict outputs step-by-step and use them as an additional input of the model to predict the next one. The details of this phase are as follows:

*Step 1:* In addition to the extracted feature vectors accompanied with the corresponding positional encoding vectors, a mostly ‘empty’ sequence Seq with only a mark for the “start-of-sequence” (Start) is given as an additional part of the model input (i.e., Seq={Start}). As a result, the model will predict the first output (i.e., arousal/valence value), which is denoted as $Output_{1}$. The element $Output_{1}$ is then appended to Seq, hence, now we have $\mathrm{Seq}=\{\mathrm{Start},Output_{1}\}$.

*Step 2:* The extracted feature vectors, accompanied with the corresponding positional encoding vectors and the updated sequence Seq, are used as the model input. As a result, the model predicts the next output $Output_{2}$ and then updates the output sequence Seq, i.e., $\mathrm{Seq}=\{\mathrm{Start},Output_{1},Output_{2}\}$.

*Step 3:* Repeat Step 2 until the “end-of-sequence” is reached, and this means the prediction is complete. In practice, we may set “start-of-sequence” and “end-of-sequence” to values outside of the ranges [−1, 1] and [0, 5] for the extended COGNIMUSE and Global EIMT16, respectively.

## 6. Experimental Results

Experiments are conducted on the expected emotion annotations in the Global EIMT16 dataset and the intended emotion annotations in the extended COGNIMUSE dataset. We compare our models’ performance based on typical evaluation metrics, namely MSE and PCC. We performed leave-one-out cross-validation for the extended COGNIMUSE dataset as mentioned in [15].

### 6.1. Proposed Model Performance and Influence of Modalities

We analyze the impact of each modality on the affective response prediction of movie viewers by using visual, audio, and text features separately as the inputs to our proposed models. The resulting MSE and PCC values on the Global EIMT16 dataset and the extended COGNIMUSE dataset are represented in Table 1, Table 2 and Table 3. We observe that the proposed models that use only audio features perform better than those using only features extracted from either the video stream or movie subtitles.

Looking at these results in more detail, for the extended COGNIMUSE dataset, the MSE and PCC of the Feature AAN are 0.125 and 0.621, respectively, for arousal, and 0.185 and 0.543, respectively, for valence, when the model input includes only audio features. Using the same Feature AAN model, the prediction accuracy is slightly worse, when the model input consists of visual features only. In particular, for arousal, the MSE and PCC are 0.152 and 0.518, respectively. These values for predicting valence are 0.204 and 0.483, respectively, for MSE and PCC.

The prediction accuracy in this case, however, is still better than when using only text features extracted from movie subtitles as the model input. The same observation can be made about Temporal AAN and Mixed AAN models. We see similar results for the extended COGNIMUSE dataset: using only audio features, our proposed models also reach higher accuracy than those with only visual features. This same observation was also observed in [21].

The higher influence of audio features may be explained by the fact that audio in movies is intentionally selected with the goal of setting an emotional context for the user. Specifically, music is useful in eliciting emotions of audiences as mentioned in [83]. Therefore, audio may have a higher impact on elicited emotions compared to video and subtitles.

We also analyze the impact of using a combination of different modalities on the performance of our proposed models. Since movie subtitles are not available in the Global EIMT16 dataset, we only examine the effect of using both visual and audio features on the models’ performance on this dataset. When visual and audio features are simultaneously used as the model input, our models perform better for both valence and arousal prediction on both datasets.

As shown in Table 4, for the Global EIMT16 dataset, the MSE and PCC of the Feature AAN for arousal are 0.742 and 0.503, respectively, and 0.185 and 0.467, respectively, for valence. For the extended COGNIMUSE dataset, these values are 0.124 and 0.630, respectively, for arousal, and 0.178 and 0.572, respectively, for valence prediction. On this dataset, our models perform best when all visual, audio, and text features are simultaneously used as shown in Table 5.

According to the results shown in Table 1, Table 2, Table 3, Table 4 and Table 5, whether the model input consists of visual, audio, or text features separately or a combination of all of them, we observe that the Feature AAN outperforms our other proposed models. When features extracted from all modalities are combined and used as the model input, the Feature AAN obtains the best MSE and PCC on the extended COGNIMUSE dataset (Table 5). Particularly, for arousal, these values are 0.177 and 0.655, respectively. For valence, they are 0.170 and 0.575, respectively. On the Global EIMT16 dataset, when using the Feature AAN for arousal prediction, we obtain 0.742 and 0.503 as MSE and PCC, respectively. These values are 0.185 and 0.467, respectively, for valence prediction (Table 4).

### 6.2. Comparison with State-of-the-Art Models

#### 6.2.1. Comparison with Baseline Models

The baseline models for the extended COGNIMUSE dataset use visual and audio features, and do not consider the movie subtitles (Table 4). When the input to the Feature AAN model includes both visual and audio features, our MSE and PCC for valence prediction are 0.178 and 0.572, respectively. These values are much better than those obtained by using the LSTM approach in [15], which was the best performing baseline model.

Notably, in [15], the authors not only use the correlation-based feature selection [127] but also apply the late fusion technique to obtain the predicted arousal and valence values. We also carried out experiments using the late fusion technique when building our models; however, our experimental results did not improve, while the training cost increased considerably.

The performance of our models is also compared with that of other baseline models [16,17,18,45,69] on the Global EIMT16 dataset. The Feature AAN with both visual and audio feature inputs outperforms the top three existing models [16,17,18] in predicting both valence and arousal values on this dataset. In particular, the MSE and PCC for arousal are 0.742 and 0.503, respectively. The values when predicting valence are 0.185 and 0.467, respectively.

Our prediction accuracy is much better than that reported in [69], and nearly equal to the one shown in [45], except for the MSE for arousal prediction. The feature vectors as well as the approach proposed in [45] are different from ours. Yi et al. [45] apply mean and standard deviation on feature vectors obtained from each movie part, and these vectors are then used as the model input, while only element-wise averaging is used in our approach.

#### 6.2.2. Comparison to Previously Proposed Models

The performance of our AAN is compared to that of our earlier models proposed in [21]. These models consist of either only fully connected layer or a two-layer LSTM structure. The same input features are used as previously mentioned in Section 3. Each of the aforementioned feature vectors is fed to each fully connected layer (of 128 neurons) of the model (with only fully connected layers) in [21]. Then, we concatenate the outputs of these layers before passing them to another two fully connected layers, whereby each layer consists of 64 neurons. According to Malandrakis’ approach [30], these layers are followed by a seven-neuron fully connected layer and a softmax layer.

The model outputs are the probabilities corresponding to the seven binned emotion responses. These discrete outputs are converted into continuous values using a low pass filter as well as the Savitzky–Golay filter [139]. These values are then rescaled into the original interval of valence and arousal values (i.e., [−1, 1] for the extended COGNIMUSE and [0, 5] for the Global EIMT16 dataset).

The LSTM structure in [21] is similar to the above described model with only fully connected layers, except that a two-layer LSTM with 64 hidden units in each layer is used instead of the two 64-neuron fully connected layers. The sequence length is also set to 5 and 4 (as mentioned in the Section 4.2 about the Temporal AAN) for the extended COGNIMUSE and the Global EIMT16 dataset, respectively.

Whether the model input consists of feature vectors extracted from video, audio, or movie subtitles separately or a combination of them (as shown in Table 1, Table 2, Table 3, Table 4 and Table 5), on both datasets, the structure with only fully connected layers performs better than the two-layer LSTM model. However, it performs worse than our proposed Feature AAN and Temporal AAN. Particularly, for the extended COGNIMUSE, when we feed all visual, audio, and text features simultaneously to the model with only fully connected layers, the MSE and PCC for arousal prediction are 0.289 and 0.229, respectively.

For the valence dimension, these values are 0.283 and 0.227, respectively, as shown in Table 5. Using both visual and audio features, the MSE and PCC for arousal are 0.989 and 0.500, respectively, for the Global EIMT16 dataset. These values for valence are 0.276 and 0.372, respectively, as mentioned in Table 4.

An ablation study is also carried out in this work, in which instead of following Malandrakis’ approach, we modify the fully connected model and the two-layer LSTM network mentioned above by replacing their last fully connected layer consisting of seven neurons by a layer consisting of only one neuron. By doing so, we now directly predict continuous values of arousal and valence.

The batch size is set to 20 for both models while the learning rate is set to 0.0001 for the two-layer LSTM network, and 0.005 for the model with only fully connected layers. We also use the loss function explained in Equation (2). The experimental results for this ablation study are shown in Table 6 and Table 7.

According to the obtained PCC and MSE, we could infer that when doing emotion prediction on the Global EIMT16 and the extended COGNIMUSE datasets, the audio features are still more influential than those extracted from other modalities. In general, the fully connected model obtains higher prediction accuracy than the two-layer LSTM. Both models outperform Malandrakis’ approach when using the visual, audio, and text features separately or their combination as the model input. However, comparison to our proposed Feature AAN, the prediction accuracy of these models is not higher.

### 6.3. Illustration of the Predicted Values

**Figure 5:**
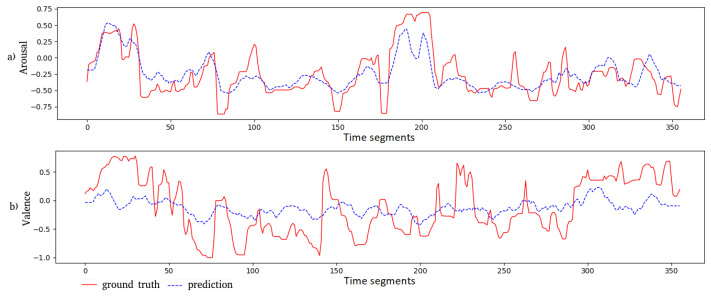
Both the ground truth and the predicted outputs of the Feature AAN model for the “Shakespeare in Love” movie clip are visualized: (**a**) for arousal and (**b**) for valence. Each time segment in the graphs corresponds to 5 s, which is also the length of each movie part.

**Figure 6:**
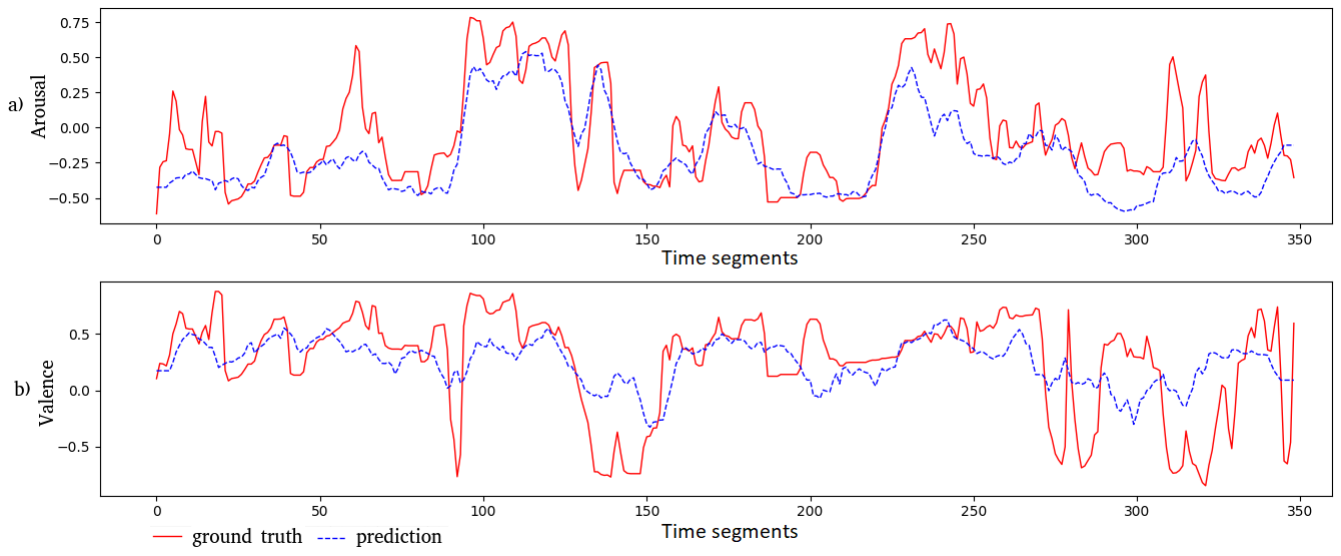
Both the ground truth and the predicted outputs of the Feature AAN model for the “Ratatouille” movie clip are visualized: (**a**) for arousal and (**b**) for valence. Each time segment in the graphs corresponds to 5 s, which is also the duration of each movie part.

We plot the predicted outputs of the Feature AAN together with the corresponding ground truth in Figure 5 and Figure 6 for a movie clip from “Shakespeare in Love” and an animated one from “Ratatouille”, respectively. The predicted arousal values and their ground truth are close to each other for both movie clips. In general, for the valence dimension, these values are less correlated. For the “Shakespeare in Love” movie, the MSE is 0.174 and 0.048 for valence and arousal, respectively. The PCC for these two dimensions is 0.649 and 0.790, respectively. For the “Ratatouille” movie clip, the MSE for valence and arousal is 0.137 and 0.068, respectively; while the PCC is 0.533 and 0.795, respectively, for valence and arousal.

## 7. Conclusions

In this work, we proposed a multimodal architecture called AttendAffectNet to predict the emotions elicited in movie viewers. Our proposed multimodal architecture was inspired by the self-attention mechanism, which was applied on both features as well as the movie’s time domain. Compared to many other studies, in which only visual and audio features are applied to predict the emotions of video viewers, in this study, we proposed a holistic approach that uses a large set of features extracted from different modalities (i.e., video, audio, and text subtitles) as the model input.

In order to obtain these features, many state-of-the-art pretrained deep neural networks and toolkits were leveraged. For the video modality, we applied the ResNet-50, RGB-stream I3D, and FlowNetS networks with parameters pretrained on the ImageNet, Kinetics, and the Flying Chairs datasets, respectively. For the audio modality, we used the OpenSMILE toolkit and the VGGish network pretrained on AudioSet. For the text modality, we applied the BERT network pretrained on Wikipedia & BookCorpus.

We proposed two methods of combining these features: self-attention across features (Feature ANN) and temporal segments (Temporal ANN). In addition, a combined approach (Mixed ANN) was also explored in this work. We performed detailed experiments on two datasets, including Global EIMT16 (for evoked emotion prediction) and the extended COGNIMUSE dataset (for intended emotion prediction). Our experiments revealed that the Feature AAN performed the best, while the Temporal AAN performed only slightly worse.

A combination of both temporal and feature components did not improve the prediction accuracy of the Mixed AAN. This might be because movie parts are long enough to deliver emotional messages, and the combined weighted effect of different modalities taken as contextual information is more relevant than the longer temporal sequence. Most importantly, all proposed AttendAffectNet variants still performed better than previous work that used the two-layer LSTM structure and a model with fully connected layers with the same features [21].

Our Feature AAN obtained a higher prediction accuracy than many other approaches in the literature and is competitive with the state-of-the-art [45]), except for a relatively higher MSE for arousal prediction. The mean and standard deviation of the extracted features obtained from each movie part were used as the model input in [45]), while only the element-wise average of feature vectors was used in our study. In future research, our approach may be further improved by using the standard deviation of the extracted features as an additional part of the model input.

A thorough analysis of the importance of different features extracted from different modalities on the resulting accuracy for emotion prediction of movie viewers was also conducted. Notably, variants of the AAN trained on audio features performed better than those trained on either visual features or text features. The reason for this might be due to the more influential impact of audio/music on evoked emotions compared with video and movie subtitles. A combination of audio, visual, and text features delivered the highest level of performance.

In this study, a wide range of features was extracted from multiple modalities. These features carry information that might be relevant for predicting the emotions of movie viewers. However, the use of many features together as the model input might also contain some redundant information. In future work, we could improve our proposed model by exploring feature selection techniques to remove the redundant features without losing much information, in addition to developing other neural network architectures.

## Figures and Tables

**Table 1:** The performance of the proposed models using only visual features.

Models (Only Video)	Extended COGNIMUSE (Intended Emotion)				Global EIMT16 (Expected Emotion)			
	Arousal		Valence		Arousal		Valence	
	MSE	PCC	MSE	PCC	MSE	PCC	MSE	PCC
**Feature AAN**	**0.152**	**0.518**	**0.204**	**0.483**	**0.933**	**0.350**	**0.764**	**0.342**
Temporal AAN	0.178	0.457	0.267	0.232	1.182	0.151	0.256	0.190
Mixed AAN	0.225	0.199	0.269	0.151	1.653	0.152	0.234	0.146
2FC-layer model	0.349	0.189	0.333	0.171	1.501	0.338	0.428	0.233
2-layer LSTM model	0.323	0.054	0.338	0.088	3.442	0.053	0.503	0.037

**Table 2:** The performance of the proposed models using only audio features.

Models (Only Audio)	Extended COGNIMUSE (Intended Emotion)				Global EIMT16 (Expected Emotion)			
	Arousal		Valence		Arousal		Valence	
	MSE	PCC	MSE	PCC	MSE	PCC	MSE	PCC
**Feature AAN**	**0.125**	**0.621**	**0.185**	**0.543**	**1.111**	**0.397**	**0.209**	**0.327**
Temporal AAN	0.162	0.472	0.247	0.254	1.159	0.185	0.225	0.285
Mixed AAN	0.219	0.204	0.269	0.160	1.650	0.290	0.235	0.314
2FC-layer model	0.299	0.203	0.299	0.173	1.533	0.395	0.368	0.318
2-layer LSTM model	0.266	0.091	0.310	0.080	2.311	0.262	0.348	0.210

**Table 3:** The performance of the proposed models using only text features.

Models (Only Subtitle)	Extended COGNIMUSE (Intended Emotion)			
	Arousal		Valence	
	MSE	PCC	MSE	PCC
**Feature AAN**	**0.175**	**0.380**	**0.237**	**0.320**
Temporal AAN	0.183	0.346	0.249	0.312
Mixed AAN	0.218	0.147	0.286	0.173
2FC-layer model	0.344	0.171	0.345	0.210
2-layer LSTM model	0.325	0.058	0.388	0.053

*Movie subtitles are not available in the Global EIMT16 dataset.*

**Table 4:** The performance of our models using both visual and audio features. FS denotes feature selection.

Models (Video and Audio)	Extended COGNIMUSE (Intended Emotion)				Global EIMT16 (Expected Emotion)			
	Arousal		Valence		Arousal		Valence	
	MSE	PCC	MSE	PCC	MSE	PCC	MSE	PCC
**Feature AAN**	**0.124**	**0.630**	**0.178**	**0.572**	**0.742**	**0.503**	**0.185**	**0.467**
Temporal AAN	0.153	0.551	0.238	0.319	0.854	0.210	0.218	0.415
Mixed AAN	0.217	0.251	0.285	0.270	1.556	0.318	0.234	0.341
2FC-layer model	0.293	0.228	0.284	0.217	0.989	0.500	0.276	0.372
2-layer LSTM model	0.247	0.083	0.301	0.092	2.222	0.254	0.303	0.208
**Sivaprasad et al. [15]**								
**(audio and video, FS)**	**0.08**	**0.84**	**0.21**	**0.50**	-	-	-	-
Yi et al. [18]	-	-	-	-	1.173	0.446	0.198	0.399
Chen et al. [17]	-	-	-	-	1.479	0.467	0.201	0.419
Liu et al. [16]	-	-	-	-	1.182	0.212	0.236	0.379
Guo et al. [69]	-	-	-	-	0.543	0.459	0.209	0.326
Yi et al. [45]	-	-	-	-	**0.542**	**0.522**	**0.193**	**0.468**

**Table 5:** The performance of our models using visual, audio, and text features.

Models (Video, Audio, and Subtitle)	Extended COGNIMUSE (Intended Emotion)			
	Arousal		Valence	
	MSE	PCC	MSE	PCC
**Feature AAN**	**0.117**	**0.655**	**0.170**	**0.575**
Temporal AAN	0.149	0.560	0.226	0.387
Mixed AAN	0.198	0.310	0.267	0.275
2FC-layer model	0.289	0.229	0.283	0.227
2-layer LSTM model	0.223	0.080	0.277	0.119

*Movie subtitles are not available in the Global EIMT16 dataset.*

**Table 6:** Predicting arousal and valence directly (without using Malandrakis’ approach): model including only fully connected (FC) layers.

Model Including Only FC Layers	Extended COGNIMUSE (Intended Emotion)				Global EIMT16 (Expected Emotion)			
	Arousal		Valence		Arousal		Valence	
	MSE	PCC	MSE	PCC	MSE	PCC	MSE	PCC
Only Video	0.186	0.426	0.247	0.372	0.999	0.308	0.477	0.207
Only Audio	0.163	0.489	0.235	0.461	0.896	0.344	0.219	0.246
**Both Video and Audio**	**0.162**	**0.503**	**0.210**	**0.498**	**0.757**	**0.478**	**0.199**	**0.418**
Only Text	0.184	0.391	0.249	0.367	-	-	-	-
**Video, Audio and Text**	**0.154**	**0.574**	**0.183**	**0.560**	-	-	-	-

**Table 7:** Predicting arousal and valence directly: The performance of the two-layer LSTM structure.

Model with 2-Layer LSTM	Extended COGNIMUSE (Intended Emotion)				Global EIMT16 (Expected Emotion)			
	Arousal		Valence		Arousal		Valence	
	MSE	PCC	MSE	PCC	MSE	PCC	MSE	PC
Only Video	0.193	0.255	0.277	0.398	1.431	0.343	0.232	0.179
Only Audio	0.167	0.483	0.241	0.422	1.413	0.340	0.231	0.289
**Both Video and Audio**	**0.231**	**0.531**	**0.285**	**0.492**	**1.354**	**0.420**	**0.228**	**0.322**
Only Text	0.197	0.220	0.263	0.210	-	-	-	-
**Video, Audio and Text**	**0.152**	**0.554**	**0.253**	**0.542**	-	-	-	-

## Data Availability

All data supporting reported results are included in this article.
